# Interpretation time for screening mammography as a function of the number of computer-aided detection marks

**DOI:** 10.1117/1.JMI.7.2.022408

**Published:** 2020-02-03

**Authors:** Tayler M. Schwartz, Stephen L. Hillis, Radhika Sridharan, Olga Lukyanchenko, William Geiser, Gary J. Whitman, Wei Wei, Tamara Miner Haygood

**Affiliations:** a Brown University, Warren Alpert Medical School, Providence, Rhode Island, United States; b University of Iowa City, Iowa City, Iowa, United States; c UKM Medical Centre, Kuala Lumpur, Malaysia; d University of Texas MD Anderson Cancer Center, Houston, Texas, United States; e Taussig Cancer Institute, Cleveland Clinic, Cleveland, Ohio, United States

**Keywords:** computer-aided detection, image perception, observer performance evaluation, technology impact

## Abstract

**Purpose:** Computer-aided detection (CAD) alerts radiologists to findings potentially associated with breast cancer but is notorious for creating false-positive marks. Although a previous paper found that radiologists took more time to interpret mammograms with more CAD marks, our impression was that this was not true in actual interpretation. We hypothesized that radiologists would selectively disregard these marks when present in larger numbers.

**Approach:** We performed a retrospective review of bilateral digital screening mammograms. We use a mixed linear regression model to assess the relationship between number of CAD marks and ln (interpretation time) after adjustment for covariates. Both readers and mammograms were treated as random sampling units.

**Results:** Ten radiologists, with median experience after residency of 12.5 years (range 6 to 24) interpreted 1832 mammograms. After accounting for number of images, Breast Imaging Reporting and Data System category, and breast density, the number of CAD marks was positively associated with longer interpretation time, with each additional CAD mark proportionally increasing median interpretation time by 4.35% for a typical reader.

**Conclusions:** We found no support for our hypothesis that radiologists will selectively disregard CAD marks when they are present in larger numbers.

## 1 Introduction

Mammography is the gold standard for breast cancer screening. However, it is not without shortcomings as a screening tool. Approximately 25% to 33% of visible cancers may be overlooked during a mammographic interpretation because of overlying dense breast tissue, variability in reader experience, and reader fatigue.^1^ To help prevent cancers from being overlooked, computer-aided detection (CAD) programs review mammograms using software designed to alert the radiologist to findings potentially associated with breast cancer, such as microcalcifications, masses, and regions of architectural distortion. CAD is notorious for creating a large number of false-positive marks.^2^^–^^4^ Its overall value in screening has been questioned, with a study in 2011 finding that in a large cohort of women undergoing screening mammography, the use of CAD decreased specificity for breast cancer detection but did not have a statistically significant improvement in detection rate or decrease in breast cancer stage, size, or nodal status.^5^ A later study by an overlapping group of authors of another large cohort of patients showed CAD to increase detection of ductal carcinoma *in situ* and to allow detection of invasive breast cancer at earlier stages.^6^ Other studies have also shown that CAD may provide some improvement in cancer detection at the expense of decreased specificity.^7^^,^^8^ Despite the mixed reviews, CAD remains in frequent use in the United States. A survey of 400 mammography sites in 2007 was followed up by surveys of the same sites in 2011 and 2016, and each time the percentage of sites using CAD for screening mammography was just over 90%, ranging from 90.2% in 2011 to 92.3% in 2016.^9^ CAD systems are also now being developed for use with digital tomosynthesis,^10^ so even though conventional mammography is becoming less frequent as a stand-alone study, information on how humans interact with CAD systems remains relevant.

The developers of ImageChecker (R2 Technology, Sunnyvale, California), a commercially available CAD system, suggested that review of CAD images would not slow the radiologist down;^11^ however, common sense would argue that review of CAD images will take a few seconds, even if CAD has marked no suspicious areas. Tchou et al.^12^ determined that review of CAD images caused a 19% increase in the interpretation time of digital screening mammograms compared with the interpretation time without CAD. Khoo et al.^1^ found that the time per reader per case was 25 s for reading film-screen screening mammograms without CAD and 45 s for reading with CAD. In both the Tchou and Khoo studies, the absolute time required to review the CAD image was nearly identical. If radiologists consider each CAD mark individually, one would expect the time required to interpret a mammogram to increase with the number of marks. Tchou et al. found that as the number of calcification or mass marks increased, so did the time taken to review them. The number of marks for masses with calcifications did not significantly affect reading times (p=0.26), perhaps because there were few such marks.^12^

Despite the findings of Tchou et al., our clinical impression has been that increased numbers of CAD marks did not lengthen the time taken by radiologists to read mammograms. In other words, we suspected that in actual clinical practice, interpretation time may not change with increasing numbers of CAD marks. This suspicion, which we developed based on our own thoughts about how we interpreted images, has also been voiced, albeit indirectly, by Fenton,^13^ who suggested that experienced radiologists may simply ignore CAD marks. Therefore, we evaluated how the time spent interpreting digital screening mammography varied with the number of CAD marks. A preliminary evaluation of the data presented here suggested that our suspicion was correct that time spent reviewing the images did not increase as the number of CAD marks increased.^14^ However, further statistical evaluation revealed a different conclusion. Therefore, we present our data here along with additional statistical evaluation.

## 2 Materials and Methods

Under institutional review board approval, we performed a retrospective review of digital screening mammograms obtained at our institution between January 1, 2011 and February 28, 2014. Patient consent was waived. We included only patients without breast implants undergoing bilateral examinations. We used only reports issued by attending radiologists who agreed to be included. The screening mammograms were viewed on Selenia digital mammography systems (Hologic, Bedford, Massachusetts). CAD images were generated using R2 ImageChecker, version 8.3.17 (R2 Technology, Sunnyvale, California), set to the manufacturer default sensitivity setting, with the microcalcifications algorithm threshold set to 2 (Algorithm v8 – increased sensitivity) and the mass algorithm threshold set to 1 (Algorithm v8 – balanced sensitivity). The Selenia units and ImageChecker were installed for screening mammography in 2007. The ImageChecker system was changed in May 2012 to Cenova Software version 1.5. This changed the appearance of the CAD image to a more processed version, but the underlying algorithms controlling the placement of CAD marks did not change. Mammograms were viewed on iSite workstations, version 3.5 (Philips Healthcare, Andover, Massachusetts/Amsterdam, The Netherlands). Radiologists entered reports on a computer-based mammography information management system (MagView, version 6.0; MagView, Burtonsville, Maryland). The workstations included two primary gray-scale diagnostic monitors (5MP Dome C5-I and E5-I; NDSsi, San Jose, California) for viewing of digital mammograms and additional color monitors for selection of comparison studies, image navigation, and supporting documents that the interpreting radiologist may have wished to consult. Workstations were equipped with display calibration software (CXtra; Dome, Waltham, Massachusetts) conforming to the part 3.14 standard of the Digital Imaging and Communications in Medicine.

We obtained completion time of each report, identity of the reader, breast density, and Breast Imaging Reporting and Data System (BI-RADS) category from the MagView database. Breast density was recorded according to the four usual types from the BI-RADS lexicon: (1) the breasts are almost entirely fatty (fatty); (2) there are scattered areas of fibroglandular density (fibroglandular); (3) the breasts are heterogeneously dense, which may obscure small masses (heterogeneous); and (4) the breasts are extremely dense, which lowers the sensitivity of mammography (extremely).

The number of CAD marks and the number of views were obtained from inspection of the images. Radiologists entered and signed reports sequentially as each study was interpreted. We defined interpretation time to be the time period between signing off of sequential reports, calculated to the nearest second. We excluded the first report of each day for each radiologist, to avoid including overnight hours between workdays that would generate extremely long apparent interpretation times. We did not exclude other specific interruptions in workflow, but did exclude outliers (defined below) from our analysis, as we believed them to be results of work interruptions. We also confined consideration to reports generated over the weekend to decrease the likelihood of interruptions and the involvement of trainees in interpretation. During weekend reading sessions, the radiologist is typically alone with no one else to initiate interruptions, and the radiologist can leave as soon as the screens have been read, which provides motivation to keep self-initiated interruptions to a minimum. Weekend screening mammography interpretation is considered to start at 3:00 p.m. on Friday afternoons and to continue through Sunday. The number of CAD marks and the number of mammogram images were determined by visual inspection of the images. Two investigators independently collected these data, one of whom correlated the results and looked at the data for the second time to resolve any discrepancies.

We calculated interpretation times before outliers or any studies, such as unilateral mammograms, were excluded. We transformed interpretation time to the logarithmic scale to achieve approximately normal linear regression model errors with constant variance. For each radiologist, transformed interpretation times that exceeded the radiologist’s median transformed reading time by more than 2.5σ^, where $\hat{\sigma }$ is the robust standard deviation estimate defined as the reader’s interquartile range divided by 1.349, were considered to be outliers and were excluded from analyses. For a normal distribution, $\hat{\sigma }$ approximates the standard deviation, but because it is computed only from the middle 50% of the data distribution, it has the advantage of not being influenced by outliers, like the conventional standard deviation estimate.

### 2.1 Statistical Analysis

Our primary goal was to determine the relationship between the number of CAD marks (CAD) and interpretation time (time) after adjustment for covariates. A linear mixed model was estimated that regressed log-transformed time on CAD, number of images, BI-RADS category, and breast density. We used the log-transformed time as the dependent variable in order that the error terms would be approximately normal. We treated both mammogram and reader as random sampling units so that conclusions would generalize to their respective populations. We did not attempt to account for correlation between mammograms from the same patient, and hence conclusions are limited to mammograms from the study patients. To account for within-reader correlation, the CAD coefficient and model intercept were allowed to vary across readers by treating them as random effects. (The term “mixed” indicates both random and fixed effects are in the model.) Using this model we estimated, for a typical reader (see Sec. 5 for definition), the percentage change in median reading time corresponding to a unit increase in CAD, i.e. an additional CAD mark as well as an interval (95% range) that contains the percentage change for 95% of the reader population for given values of the other covariates. We also estimated the percentage change in median reading time corresponding to a unit increase in each of the other predictor variables, which we assumed to be the same across readers.

The precise statistical specification of the mixed linear regression model, as well as formulas for deriving the percentage change for a typical reader and the 95% range across readers, is provided in Sec. 5. We used the SAS procedure MIXED^15^ to estimate the mixed regression model and to assess the usefulness of each predictor. All tests were two-sided, with p values <0.05 considered statistically significant.

## 3 Results

Ten radiologists participated. All were certified by the American Board of Radiology and practice screening mammography. The median experience after residency for these radiologists was 12.5 years (range 6 to 24 years). These radiologists interpreted 1945 eligible screening mammograms taken from a total of 1829 patients: 1719 patients contributed a single mammogram, 104 patients contributed 2 mammograms, and 6 patients contributed 3 mammograms. Each radiologist reviewed an independent set of mammograms; thus, no two readers reviewed the same mammogram. After excluding outliers, 1834 mammograms remained. Two more mammograms that were the only mammograms having a BI-RADS score of 3 were also dropped, leaving a total of 1832 mammograms that were used for the analysis.

**Fig. 1:**
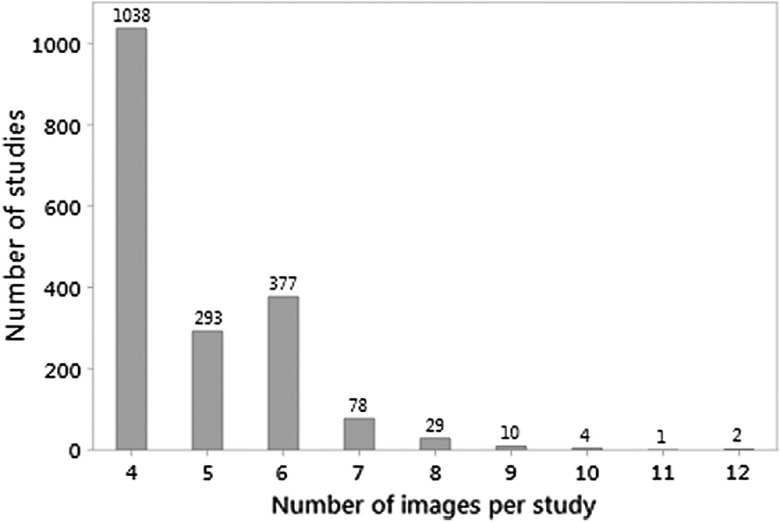
Number of mammographic studies by the total number of images obtained per study.

**Fig. 2:**
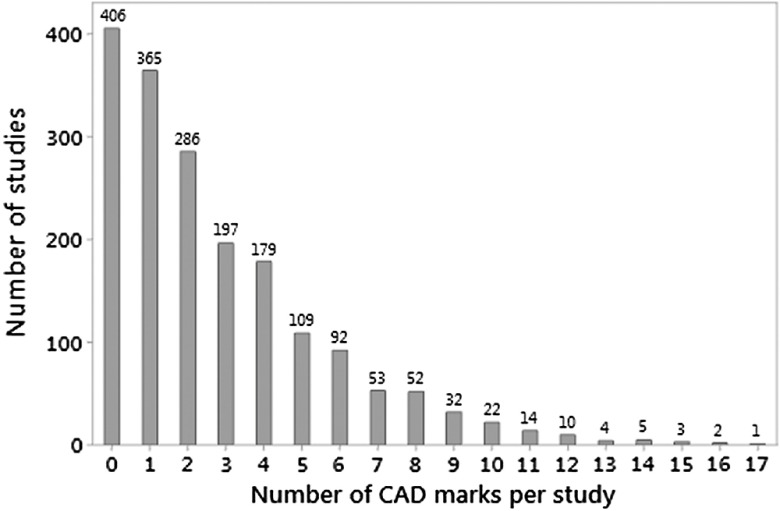
Number of mammographic studies by the number of CAD marks generated.

As shown in Fig. 1, for the 1832 mammographic studies, the number of images obtained for each mammography study ranged from 4 to 12 (median = 4), with 1038 (56.7%) having four images, which consisted of the standard two mediolateral oblique and two craniocaudal images. Two hundred and ninety-three studies (16.0%) had five images. Only two studies (0.11%) had 12 images. The number of CAD marks per study ranged from 0 to 17 (median = 2). As shown in Fig. 2, the number of CAD marks per study was positively skewed. Four hundred and six of the 1832 studies had no CAD marks (22.2%), and there were relatively few studies with very high numbers of CAD marks, although one study had 17 CAD marks. A summary of the number of CAD marks and images is available in Table 1.

**Table 1:** Statistical summary of numbers of images, CAD marks, triangles, and asterisks per mammographic study.

Statistic	Images	CAD marks	Triangles	Asterisks
Mean	4.82	2.8	1.37	1.39
Minimum	4	0	0	0
25^th^ Percentile	4	1	0	0
Median	4	2	0	1
75^th^ Percentile	6	4	2	2
Maximum	12	17	13	12

On the CAD images, triangles are marks denoting possible malignant microcalcifications, and asterisks denote possible malignant masses. Total sample size is n=1832 studies.

We examined how interpretation time and the covariates related to the number of CAD marks. Table 2 presents the mixed linear regression results. From Table 2, we see that CAD, number of images, and BI-RADS are all statistically significant predictors of ln(time) (all p≤0.0001), but density did not attain statistical significance (p=0.58). Table 2 presents corresponding coefficient estimates for CAD and number of images, as well as for each level of the categorical predictors BI-RADS and density. Because breast density did not attain statistical significance, to simplify interpretation of the results, we have omitted corresponding coefficient estimates from Table 2.

**Table 2:** Linear mixed model results.

Predictor variable	Coefficient [95% CI]	SE	p value	df	% Change in time [95% CI]
Number of CAD marks	0.0426 [0.028, 0.058]	0.0066	0.0001	1	4.35 [2.80, 5.92]
Number of images	0.0835 [0.060, 0.108]	0.0122	<0.0001	1	8.71 [6.13, 11.35]
BI-RADS (ref = 2)			<0.0001	2	
(level = 0)	0.1920 [0.110, 0.276]	0.0419	<0.0001	1	21.1 [11.6, 31.5]
(level = 1)	−0.1980[−0.260,−0.136]	0.0317	<0.0001	1	−18.0[−22.9,−12.7]
Breast density			0.5800	3	

The dependent variable is ln(interpretation time). For the number of CAD marks and number of images, the percentage change in time is for each unit increase in the number of CAD marks or number of images. Coefficient estimates are not shown unless the predictor variable was statistically significant. CI = confidence interval; BI-RADS = categorical BI-RADS score (0, 1, or 2), with 2 as the reference level; Density = categorical density score (“extremely,” “heterogeneous,” “fibroglandular,” or “fatty”); df = degrees of freedom; df = 1 indicates a single coefficient test; df≥2 indicates a global test that tests if there are any differences among (df + 1) categorical variable level effects; SE = standard error.

From Table 2, we see that predicted ln(time) increases by 0.0426 for each unit increase in CAD, i.e., for each additional CAD mark. A more natural interpretation is that, for a typical reader, interpretation time increases proportionally by 4.35% (CI = 2.80%, 5.92%) for each additional CAD mark, as shown in the last column. In addition, we estimate that for 95% of the reader population, the increase in interpretation time ranges between 1.74% and 7.03% (not shown in Table 1). (Section 5 demonstrates how these proportional-change results are computed.) By comparison, the increase is 8.71% in interpretation time for each additional image. BI-RADS codes 0 and 1 resulted in an increase of 21.1% and a decrease of 18.0%, respectively, compared to the interpretation time for BI-RADS code 2, the reference code.

**Fig. 3:**
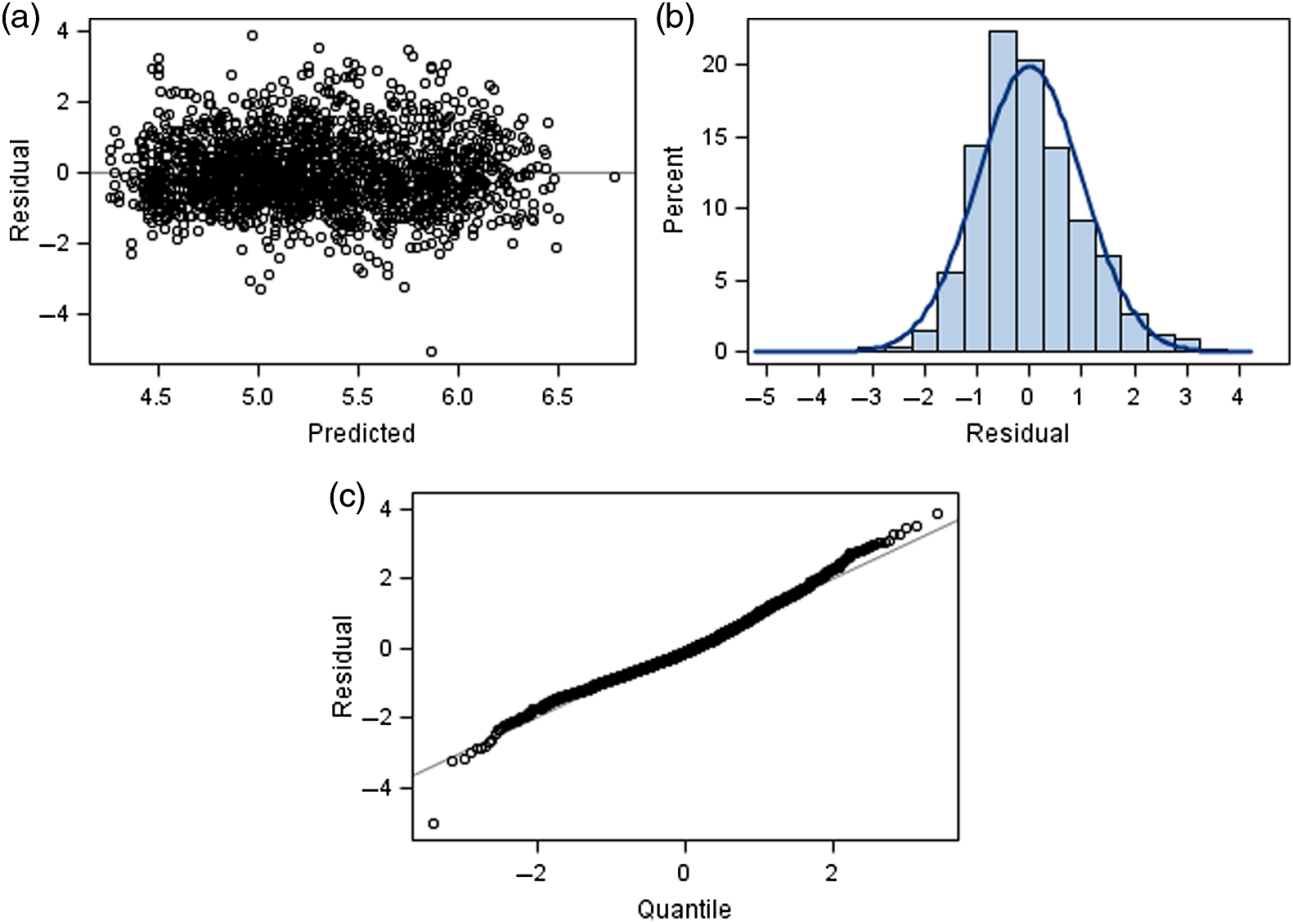
Conditional studentized residual plots. (a) Residual versus predicted. (b) Histogram of residuals. (c) Residuals versus normal quantiles.

We also tested for a nonlinear (quadratic) effect of CAD, as well as for interactions between CAD and the covariates, none of which attained statistical significance. A plot of the conditional studentized residuals versus the predicted values [Fig. 3(a)] suggested fairly constant variance of the error terms and no obvious model misspecification. (A studentized residual is equal to the residual divided by an estimate of its standard deviation.) A plot of the conditional studentized residuals versus normal quantiles [Fig. 3(c)] and a histogram of the conditional studentized [Fig. 3(b)] suggested that the error terms were approximately normally distributed.

## 4 Discussion

We studied the time taken by radiologists during actual clinical interpretation of screening mammograms and modeled the logarithmic transformation of interpretation time as a linear function of the number of CAD marks on the images, number of images, BI-RADS score, and density. We found that as the number of CAD marks increased, the predicted interpretation time also increased, increasing proportionally by 4.35% for each additional CAD mark for a typical reader, assuming that number of images, BI-RADS score, and density remain constant. For example, our findings imply that for a typical reader, if the median interpretation time for mammograms without any CAD marks is 120 s, then for mammograms with one CAD mark the median interpretation time would be 125.2 s. This result did not support our subjective impression that additional CAD marks were not associated with additional interpretation time. Although we cannot say with certainty that the extra time taken for mammograms with CAD marks was actually dedicated to studying the CAD marks, our findings also do not support a suggestion by Fenton^13^ that many radiologists ignore CAD output.

Rather, our findings support those by Khoo et al.^1^ and Tchou et al.^12^ that more CAD marks require more interpretation time. Our study also complements the study by Tchou as it was performed prospectively, with an observer watching interpretation and recording time taken for interpretation without CAD and then the time needed to incorporate the CAD results. The study design in the paper by Tchou et al. may have influenced the outcome by making the radiologists self-conscious as well as by adding a step to their workflow. Our study was retrospective and therefore there was no possibility that the research itself would influence the behavior of the radiologists.

We note that the failure of an earlier analysis^14^ of the same data to find a significant association between interpretation time and number of CAD marks can be attributed to low power attributable to only testing individually if there was a significant association for each reader, rather than fitting a model based on the data from all of the readers, as was done in this study.

Our study had some limitations. There were relatively few studies with a large number of CAD marks. Therefore, our results may be more reliable for differences in reading times between studies with small or moderate numbers of marks rather than large numbers. As this study was retrospective, we could not deliberately or specifically exclude the studies with common, short workflow interruptions, e.g., brief phone calls. These interruptions, however, were likely random with respect to the number of CAD marks, and many may have been excluded as outliers. Our results may also have been different if we had included interpretations performed during the workweek. This would have increased the number of studies available for inclusion but would have caused more variation in apparent interpretation time due to work-related consultations and interruptions. For example, during the workweek, our radiologists often read screening mammograms in between other examinations, such as ultrasound or between procedures. These studies are not entered into MagView and therefore the time taken with such studies would have been spuriously added to the apparent interpretation time of the next screening study. On the weekends, however, on-call radiologists come in specifically to interpret screening mammograms performed Friday afternoon, and the radiologists will not have other types of imaging to interpret. For these reasons, we believed that it was best to confine our consideration to weekend interpretations. We studied interpretation times of a specific group of radiologists using one specific type of CAD program and may have had different results had we studied others. Finally, we note that although conclusions from this study generalize to both the reader and mammogram populations, the mammogram population is restricted to mammograms generated from subjects in this study. However, we do not consider this to be an important limitation because (1) our interest was primarily in reader behavior and (2) the number of subjects was relatively large.

In summary, our data suggest that radiologists do spend extra time evaluating studies with more CAD marks. Therefore, we found no support for suggestions that CAD marks are ignored. Our findings also support the commonsense assumption that a reduction in the number of false positive CAD marks would be helpful to improving workflow in screening mammography. This supports the efforts being made to use artificial intelligence to reduce the number of false-positive marks.^16^

## 5 Appendix A: Statistical Details, Results, and Examples

### 5.1 Specification of the Mixed Linear Regression Model

In this section we describe in statistical detail the mixed regression model used to estimate the effect of CAD on the logarithm of interpretation time. In this model the intercept and CAD coefficient are allowed to vary across readers to account for within-reader correlation.

Each reader reads a different set of mammograms. For reader i reading the j’th mammogram (among those assigned to reader i), the model is given by

$$\ln({\text{time}}_{ij})=\beta_{0i}+\beta_{1i}({\mathrm{CAD}}_{ij})+\beta_{2}({\text{images}}_{ij}-4)+\beta_{3}{(\mathrm{BI}-\mathrm{RADS}}_{ij}=0)\;+\beta_{4}{(\mathrm{BI}-\mathrm{RADS}}_{ij}=1)+\beta_{5}{(\text{density}}_{ij}=“\text{Extremely}”)\;+\beta_{6}{(\text{density}}_{ij}=“\text{Heterogeneous}”)+\beta_{7}{(\text{density}}_{ij}=\text{“}\text{Fibroglandular}”)+\varepsilon_{ij},$$ (1)

where “ln” is the natural logarithmic function; $\beta_{0i}$ is the intercept term for reader i; ${\mathrm{CAD}}_{ij}$ is the number of CAD marks with corresponding reader-specific coefficient $\beta_{1i}$; ${\text{images}}_{ij}$ is the number of images; ${\mathrm{BI}\text{-}\mathrm{RADS}}_{ij}$ is the BIRADS category where, e.g., ${(\mathrm{BI}\text{-}\mathrm{RADS}}_{ij}=0)$ is an indicator variable that takes the value 1 if ${\mathrm{BI}\text{-}\mathrm{RADS}}_{ij}=0$ and otherwise is 0; ${\text{density}}_{ij}$ is the breast density category; and $\varepsilon_{ij}$ is the error term. We assume that the $\left(\beta_{0i},\beta_{1i}\right)$ pairs have a bivariate normal distribution across readers with means $\beta_{0}$ and $\beta_{1}$, variances $\sigma_{\beta_{0i}}^{2}$ and $\sigma_{\beta_{1i}}^{2}$, and covariance $\sigma_{\beta_{0}\beta_{1}}$, and we assume that the $\varepsilon_{ij}$ are independent and normally distributed with zero mean and variance $\sigma_{\varepsilon }^{2}$. Note that only the intercept and CAD coefficient vary across readers; i.e., for the other independent variables, the corresponding regression coefficients are assumed constant across readers. Because all mammograms had at least four images, the variable $\left({\text{images}}_{ij}-4\right)$ is used to simplify the interpretation of the intercept. Note that Eq. (1) treats BI-RADS category 2 and breast density category “fatty” as the reference levels by omitting them.

### 5.2 Regression Model Estimates and Diagnostic Plots

The fitted regression model corresponding to Eq. (1) is given by

ln(time)=5.108+0.043(CAD)+0.084(images−4)+0.192(BI−RADS=0)−0.198(BI−RADS=1)+0.063(density=“Extremely”)+0.056(density=“Heterogeneous”)+0.084(density=“Fibroglandular”).

### 5.3 Computation and Estimation of Proportional Change in Interpretation Time and 95% Range

**Result 1: Proportional change.** Under the assumptions of the mixed linear regression model described in Eq. (1), it follows that each exponentiated slope coefficient minus 1 is the proportional change in the median interpretation time attributed to an increase of one unit in the corresponding independent variable with the other independent variables remaining constant. For the CAD predictor, this result is for a typical reader. (Reader i is defined as a typical reader if its CAD coefficient is equal to the mean CAD coefficient, i.e., if $\beta_{1i}=\beta_{1}$.)

Proof. We first show the result for a one unit change in CAD. Consider a typical reader $\left(\beta_{1i}=\beta_{1}\right)$ and let the vectors Z and B denote the other independent variables (including intercept) and corresponding parameters, respectively. It follows for the typical reader, conditional on Z=z, that the expected value of the log-transformed time is given by $E[\ln(\text{time})]=\beta_{1}(\mathrm{CAD})+{\text{B}}^{\prime }z$. Because the model assumption of normal error terms implies that ln(time) is normally distributed, conditional on the covariates, it follows that $\beta_{1}(\mathrm{CAD})+{\text{B}}^{\prime }z$ is also the median ln(time), and hence $\exp[\beta_{1}(\mathrm{CAD})+{\text{B}}^{\prime }z]$ is the median of the nontransformed interpretation time. Thus, the ratio of the median interpretation times corresponding to an increase of one unit in CAD is given by

$$\frac{\exp[\beta_{1}(\mathrm{CAD}+1)+{\text{B}}^{\prime }z]}{\exp[\beta_{1}(\mathrm{CAD})+{\text{B}}^{\prime }z]}=\exp(\beta_{1})$$

and the proportional change by

$$\exp(\beta_{1})-1.$$ (2)

Note that this result does not depend on either the number of CAD marks or the values of the other covariates. The percentage change is $100[\exp(\beta_{1})-1]$. The proof for the other covariates is similar, except that the result applies to all of the readers, not just a typical reader.

#### 5.3.1 Example

The estimate for $\beta_{1}$ (see Table 2) is 0.04256. Plugging this value into Eq. (2) results in

exp(0.04257)−1=0.04349. (3)

Thus, we estimate that there is a 4.34% increase in the median interpretation time with each additional CAD mark for a typical reader. Lower and upper 95% confidence interval bounds for the proportional-change result from replacing $\beta_{1}$ in Eq. (2) by the 95% confidence interval upper and lower bounds for the $\beta_{1}$ estimate. For example, from Table 2, the 95% confidence interval for $\beta_{1}$ is [0.028, 0.058]; exponentiating both endpoints and subtracting one yields the 95% confidence interval [0.0280, 0.0592] for the proportional change, or equivalently [2.80%, 5.92%], as given in Table 2.

**Result 2: 95% range.** Under the assumptions of the mixed linear regression model described in Eq. (1), it follows that for 95% of the reader population the proportional change in the median interpretation time attributed to an increase of one unit in CAD will be within the interval

$$[\exp(\beta_{1}-1.96\sigma_{\beta_{1i}})-1,\;\exp(\beta_{1}+1.96\sigma_{\beta_{1i}})-1].$$ (4)

Proof. The proof is similar to that for Result 1 for CAD, with the typical reader being replaced, in terms of the CAD coefficient distribution across readers, by the 2.5th percentile ($\beta_{1i}=\beta_{1}-1.96\sigma_{\beta_{1i}}$) and the 97.5th percentile ($\beta_{1i}=\beta_{1}+1.96\sigma_{\beta_{1i}}$) readers to obtain the lower and upper boundaries, respectively, of Eq. (4).

#### 5.3.2 Example

We estimate the boundaries of the interval Eq. (4), by replacing parameters by their estimates. For example, using the regression model estimates β^1=0.04257 and σ^β1i2=0.000167, the estimated interval is

$$[\exp(0.04257-1.96\sqrt{0.000167})-1,\exp(0.04257+1.96\sqrt{0.000167})-1]\;=[0.0174,0.0703]=[1.74\%,7.03\%].$$ (5)
